# Pool Boiling Heat Transfer Characteristics of SiO_2_ and BN Nanoparticles Dispersed Mono and Hybrid Nanofluids

**DOI:** 10.3390/nano13192625

**Published:** 2023-09-23

**Authors:** Wagd Ajeeb, S M Sohel Murshed

**Affiliations:** IDMEC, Instituto Superior Técnico, University of Lisbon, 1049-001 Lisbon, Portugal

**Keywords:** pool boiling, boron nitride, silica, critical heat flux, burnout heat flux, hybrid nanofluids

## Abstract

This study reports an experimental investigation of pool boiling (PB) heat transfer performance of hybrid (two types of particles) and mono (single-particle) nanofluids consisting of Boron nitride (BN) and Silicon dioxide (SiO_2_) nanoparticles (NPs). While hybrid nanofluids (HNFs) were prepared in a total particle concentration of 0.05 vol.% with four different percentages of these two types of NPs (are 0.01/0.04, 0.02/ 0.03, 0.03/0.02, and 0.04/0.01 (BN vol.%/SiO_2_ vol.%)), two mono nanofluids (MNFs) of BN and SiO_2_ nanoparticles were prepared at the same total concentration of 0.05 vol.% for each NP type. Both nanofluids (NFs) were prepared in the base fluid (BF), which is the mixture of 15 vol.% of ethylene glycol (EG) and 85 vol.% of distilled water (DW). Then, the boiling heat transfer performance of these MNFs and HNFs was assessed by experimentation in a pool boiling test rig. The obtained results demonstrated good improvements in critical heat flux (CHF) and burnout heat flux (BHF) of both types of NFs. The CHF increased by up to 80% for BN-based MNF and up to 69% for HNF at 0.04 vol.% BN, which is the maximum percentage of BN into HNF, while the lowest improvement in CHF was 48% for the SiO_2_-based MNF compared to the BF. Similarly, the BHF was found to increase with the increasing in the loading of BN nanoparticles and a maximum enhancement of BHF of 103% for BN-based MNF was observed. These HNFs and MNFs exhibited significantly improved pool boiling heat transfer performance compared to this BF, and it became lower by increasing the percentage of SiO_2_ NPs in the HNFs.

## 1. Introduction

The development of advanced thermal management processes and systems became urgent in many sectors such as in electronics, chemical industries, and transportation sectors. The heat exchange systems various geometrical designs are developed to enhance their thermal performance, and most of the heat exchangers are increasingly failing to adequately meet the thermal management demand of those application sectors [1,2]. One important heat transfer process, which is the phase change of fluids, is used in systems containing boiling and condensing, such as the heat pipe. The boiling technique exists in the most important industrial applications for thermal management, such as in nuclear systems. Despite having very high heat transfer, the bubbles’ creation and their dynamics in boiling require a complex understanding of this phenomenon [3]. However, the use of conventional thermal fluids with their limited properties limited the development of the boiling process to smaller and more effective phase-change-based heat exchangers for various applications [4,5,6]. New thermal fluids called nanofluids (NFs) have appeared widely from the beginning of this century, and a large number of studies found their enhanced thermal properties and improved heat transfer features, such as a higher heat transfer coefficient [7] and better thermal performance of the heat exchangers and boiling systems [8]. Thus, adding the NPs to the conventional thermal fluids can play a significant role in increasing the CHF in the boiling process, leading to better cooling for the high-heat-producing equipment and systems with lower temperatures for process safety and device protection [9]. The boiling performance was discovered to be enhanced by using NFs in many studies [10,11], and several recent studies have been conducted to assess the boiling behavior of various NFs [12]. For example, the use of Al_2_O_3-_dispersed MNF in the PB of in-vessel retention was shown to improve the CHF during the boiling period [13]. Developments in the NF field allowed the discovery of new possible types of NFs, such as HNFs, by combining two types of NPs in their preparation, providing more advantages for their adjustable and advanced thermophysical properties [14]. Research on HNF aims to boost and optimize the enhancement of each MNF by the potential interactive impacts between the different types of NP materials [15]. Kumar et al. [16] studied MWCNT-SiO_2_ HNF in car-radiator-based EG/DW as a coolant, and an improvement of about 40% in the temperature drop and an increase in cooling power were reported. Also, Ma et al. [17] reported the performance of graphene–silver HNFs in a PB system at three particle concentrations. Their results showed an enhancement of the CHF of up to 52.31% compared to the BF. The thermal performance of the boiling of HNF consisting of 50% Al_2_O_3_ and 50% CeO_2_ NP-based DW in a horizontal tube was assessed by Kamel et al. [18]. The results reported an enhancement ratio of up to 1.37 compared to the DW, and the high heat flux was removed for the small temperature difference. The same group [19] also studied the boiling of an aqueous HNF consisting of GNP (graphene nanoplatelets) and Fe_3_O_4_ NPs and compared its performance with other types of NFs, mainly GNP MNF, GNP-Fe_3_O_4_ +Al_2_O_3_ HNF, and GNP-Fe_3_O_4_ +SiO_2_ HNF. Their results showed considerable enhancements in heat transfer, and GNP-Fe_3_O_4_+Al_2_O_3_ HNF and GNP-Fe_3_O_4_+SiO_2_ HNFs had the best performance, with enhancements of up to 82% and 92% compared to DW, respectively. Reddy et al. [20] studied the thermal properties and CHF of DW-based Al_2_O_3_ and CuO HNF. They reported good improvements of thermal conductivity, by 15% and 49% in CHF at 0.1 vol. % compared to those of the base fluid, i.e., DW. Huang et al. [21] investigated the boiling performance of GNPs-SiO_2_ HNF-based DW. The boiling heat transfer coefficient (BHTC) was found to reduce, and it was assumed to be due to an increase in particle concentration and the changing of surface roughness. However, they reported a reduced boiling heat transfer performance due to raising the surface tension. Sharma et al. [22] studied the boiling performance of Ag/ZnO HNF over a heater surface modified by electro-discharge machining (EDM). The 0.1 vol. % of Ag/ZnO NPs over a particular surface (E3) showed the highest CHF and BHTC. The same group [23] also studied Ag/ZnO HNFs in boiling and demonstrated that surface roughness and particle concentrations have a significant impact on the boiling performance, and found an increase in BHTC of about 250% and in CHF of about 80% compared to the BF.

However, there is still a need to further understand the thermal behavior of boiling NFs in general, as well as to discover new advanced NF types with low NP concentrations and superior thermal properties. Furthermore, the previous studies on the performance of HNFs and their complex mechanisms in PB are not explored well in terms of their phenomena. Also, many new nanomaterials which have very good stability and thermal properties have still not been studied in HNF preparation, and neither has the assessment of their performance under boiling conditions such as HNFs. Therefore, this study intends to prepare new promising types of MNFs and HNFs (of BN and SiO_2_ HNFs at low NPs concentration) that have not yet been studied the literature (to the best of the authors’ knowledge), and to investigate their thermal features and performance in bool boiling applications and compare them with their MNFs and the correlated BF at the same particle concentration.

## 2. Preparation of Nanofluids

**Figure 1:**
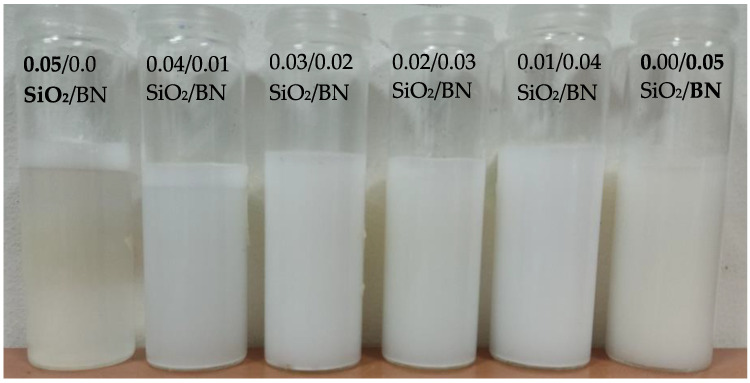
MNF and HNF samples of different combinations of concentrations (vol.%/vol.%).

The nanofluid samples were prepared at one total volumetric concentration (0.05 vol.%) for each sample. Two types of NPs which were used to prepare the MNFs and HNFs are BN (about 70 nm in size with a purity of 99%, as provided by the supplier- Iolitec Nanomaterials, Germany) and SiO_2_ (about 20 nm in size with a purity of 99%, as obtained from the same supplier—Iolitec Nanomaterials, Germany). The nanoparticles’ concentration in HNFs was set to be 0.05 vol.% with four different percentages of these two NPs (0.01/0.04, 0.02/0.03, 0.03/0.02, and 0.04/0.01 (BN vol.%/SiO_2_ vol.%)). In addition, two MNF samples—one with the BN NP and the other with the SiO_2_ NP—were prepared at 0.05 vol.% concentration for each. The base fluid (BF) for both MNFs and HNFs was EG/DW mixture at 15/85 (vol.%/vol.%) for all the samples. The selection of the aqueous EG/DW comes from their common usage in industrial applications for heating and cooling [24,25,26]. Each sample of 0.5 liter was prepared as needed for running the experiment. After determining the weight of each type of NP to obtain the desired concentration, the dispersion of the NPs into the BF was performed first by mixing for 20 min via a magnetic stirrer, then a sonication process for 25 min at 40 kHz frequency using an ultrasonicator (UP200Ht from Hielscher, Germany). The latter enabled good homogenization and stability of the resulting NFs. The prepared MNF and HNF samples are shown in Figure 1.

The colors of the samples refer to the materials of the NPs used, and the color gradient changed according to the concentration for each type of NP in the HNFs. Also, each sample had a uniform color between the top and the down of the vial which confirms the good depression and stability of the NFs. Although stability of nanofluids is critical for their thermal properties, like thermal conductivity, it is, however, not crucial in the boiling experiments due to the fact that during boiling, nanofluids undergo vigorous agitation due to bubbles’ nucleation and their upward flow.

## 3. Measurement of Thermal Conductivity

**Figure 2:**
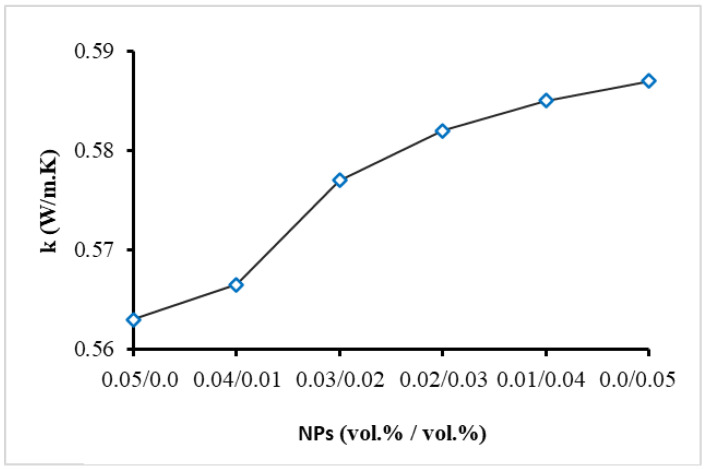
Thermal conductivity of MNFs and HNFs.

Thermal conductivity has been considered a key thermal property for NFs’ performance in thermal management applications. The thermal conductivity of the MNFs and HNFs was measured at room temperature (19 °C) using the Thermal Properties Analyzer from METER, USA, which works on the popular transient hot-wire method that has been commonly used for NFs and thus is not detailed here. Several measurements for the MNFs and HNFs were conducted considering a break time of 25 min. Furthermore, a calibration process of this device was performed using the BF, and the highest deviation was found to be around ±1.4% compared to the standard reference. The obtained experimental results for the thermal conductivity of the MNFs and HNFs are demonstrated in Figure 2, which shows reasonable enhancements in thermal conductivity for all NFs in comparison with the BF (EG/DW 15/85 (vol.%/vol.%)). Thermal conductivity was increased by the rising NP percentage of BN NP type (until a maximum of 0.05 vol.%) by up to about 7% for BN MNF. The higher thermal conductivity of the BN material compared to the SiO_2_ material causes this increasing trend of thermal conductivity for HNFs by increasing the percentage of BN NPs. It can also be observed from Figure 2 that there is relatively less increase in the thermal conductivity at 0.01/0.04 (BN/SiO_2_), and then higher values appear with increasing the BN percentage. The latter can be due to the smaller effect of BN NPs’ motion and collisions at this concentration (0.01 vol.%), hence the smaller impact on enhancing the thermal conductivity of the HNF. For the HNFs, the enhancement in thermal conductivity was increased from 3.2% at 0.01/0.04 (BN/SiO_2_) to 6.5% at 0.04/0.01 (BN/SiO_2_).

Also, the thermal conductivity of SiO_2_ MNF was found to increase by about 2.5% in comparison with the BF. Nevertheless, SiO_2_ NPs are available in abundance at relatively cheap prices compared to BN NPs, and thus their impact on thermal conductivity enhancement (such as 2.5% for SiO_2_ MNF) at such low concentration is noteworthy for cooling and heating applications.

## 4. Experimental Setup and Measurements

**Figure 3:**
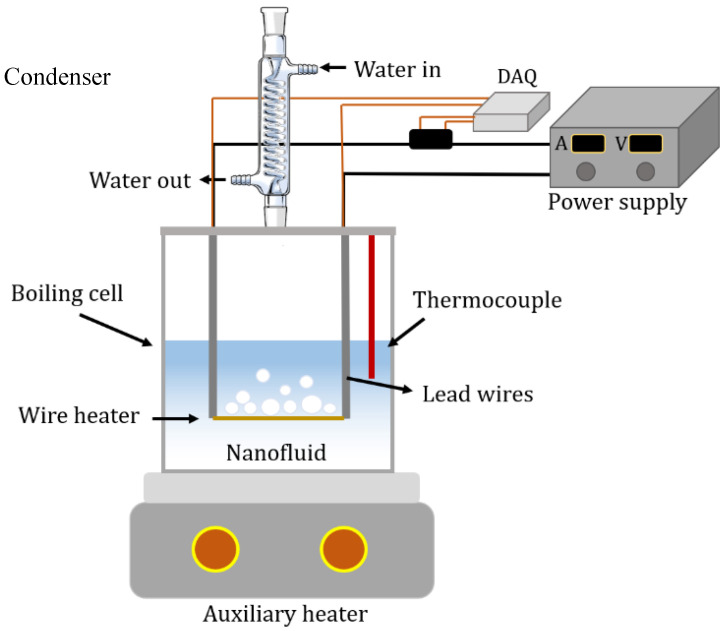
The schematic of pool boiling setup [27].

The experimental setup was previously developed and used for the pool boiling (PB) heat transfer performance of both HNFs and MNFs. The schematic of the setup is provided in Figure 3. Detailed information about the PB setup and operation conditions was presented in a prior study [27]. The accuracy of the PB setup was primarily checked and calibrated with DW, in addition to validating against relevant work in the literature under the same operation conditions [27]. The setup contained a heater located under the boiling chamber, a condenser on the top of the boiling chamber, a power supply, K-type thermocouples, and a data acquisition system. The NF samples were taken in the glass chamber for boiling with the possibility of bubble visualization. Furthermore, a horizontal wire made of Nickel-Chromium (Ni-Cr) was selected as a heating element, with dimensions of 4.27 cm long and a diameter of 250 micrometers.

The HNFs and MNFs were initially heated in the chamber until the saturation temperature of the BF. This is because the saturation temperatures of the NFs were not known or available. By increasing the voltage provided by the power supply that is applied to the heating wire, the electrical current increased too, and the recorded data were collected. The calculation of the electrical resistance (*R* = *V*/*I*) of the wire was carried out based on the values of the electrical current (*I*) and voltage (*V*). Then, the wire temperature (T_w_) was obtained by the calibration curve established for the relation between the temperature and the electrical resistance in the previous study [27], which followed the approach used in similar studies in the literature [11,28].

Therefore, the boiling curve of each sample of the MNFs and HNFs was established through the heat flux (*q*) and the wall superheat, i.e., *T_w_* − *T_sat_*, where *T_sat_* is the saturation temperature which is also confirmed via a thermocouple. Then, the acquired PB curve for each sample of the HNFs and MNFs was analyzed to determine the CHF value. The heat flux (q) was calculated using supplied electric power by Equation (1):

(1) $$q=\frac{VI}{\pi dL}$$

where *d* and *L* are the diameters and the length of the heating wire, respectively. Moreover, the boiling heat transfer coefficient (HTC) of the HNFs and MNFs for the nucleate boiling regime was determined by the following equation of modified Newton’s cooling law:

(2) $$h=\frac{q}{T_{w}-T_{s}}$$

## 5. Boiling Results and Discussion

**Figure 4:**
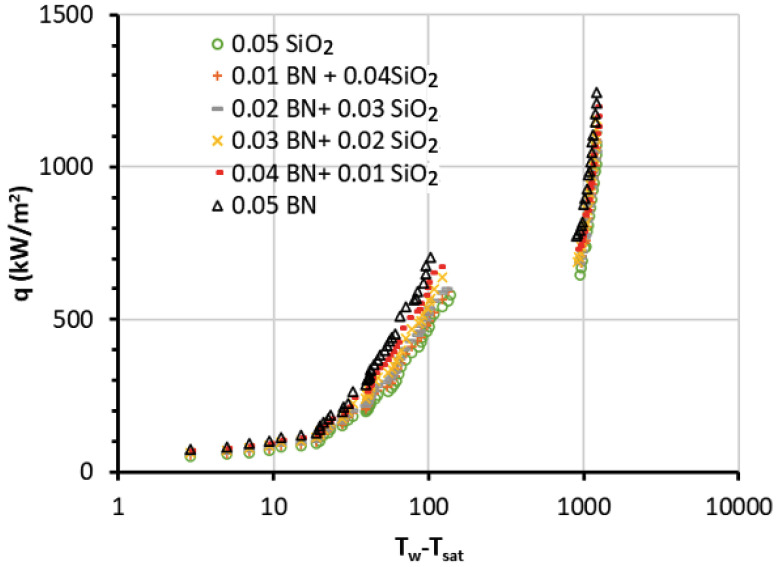
The pool boiling curves for the MNFs and HNFs.

The experimental tests for the NFs in the PB system were carried out for each sample at atmospheric pressure. The results were gathered to find out the values of the heat fluxes (*q*) at the corresponding wall superheat (*T_w_ − T_sat_*) for all NFs, as well as BF. The pool boiling curves for the MNFs and HNFs are shown in Figure 4. The resulting boiling curves follow the typical theory of boiling, where it starts with natural convection at low superheat, followed by the nucleate boiling regime and transition regime, and ends with the film boiling stage. However, the impact of the wire surface in the case of NF boiling can be different from the use of conventional fluids due to the NP deposits on the surfaces, which may cause random microstructures. The accurate estimation of the boiling curve and the heat transfer mechanisms needs the determination of the surface characteristics, such as the nucleation sites with cavity, and the pattern allocation, which can be difficult in the case of NFs and their complex forms, such as HNFs at nano/micro levels.

In this study, the data denote that by increasing the BN concentration in the HNF, the boiling curve goes to the left, resulting in the nucleate regime and thus the CHF occurring earlier. Additionally, the CHFs of HNFs and MNFs were found to be higher than the ones for BF and increased by increasing the BN percentage and decreasing the SiO_2_ in the HNF. The data of the MNFs showed improvements in CHF of about 80% for BN MNF and 48% enhancements for SiO_2_ MNF at 0.05 vol.% compared to the BF. On the other hand, the data of the HNFs indicated an improvement in CHF of up to 69% for BN/SiO_2_ of 0.04/0.01 and 51% for BN/SiO_2_ of 0.01/0.04.

**Figure 5:**
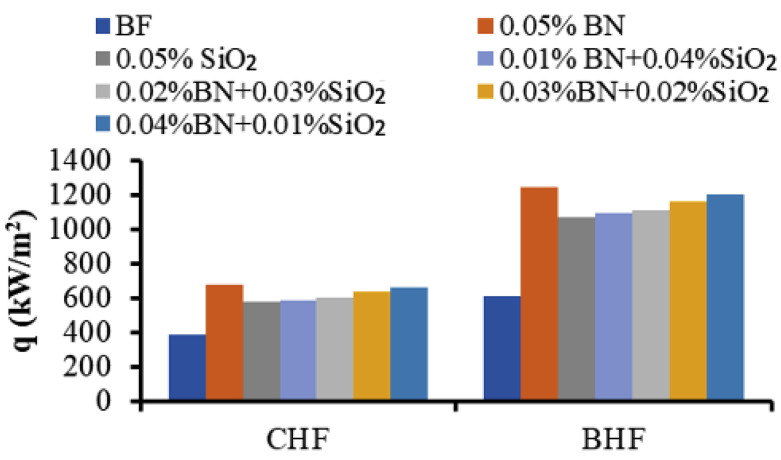
CHF and BHF levels for the MNFs and HNFs.

In addition, the burnout heat flux (BHF) for the MNFs and HNFs can be determined by analyzing the pool boiling curves and the maximum point (heat flux) of each curve where the wire was burnt. The BHF results were collected and compared with the CHF results, as shown in Figure 5. The improvements of the BHF were about 103% for BN MNF and 75% for SiO_2_ MNF. Accordingly, the BHF points of the MNFs and HNFs were increased in a similar way to the CHF points by increasing the concentration of BN NPs in the HNF and decreasing the concentration of SiO_2_ NPs. However, BHF points demonstrated higher enhancements than the CHF points in comparison to the related BF.

**Figure 6:**
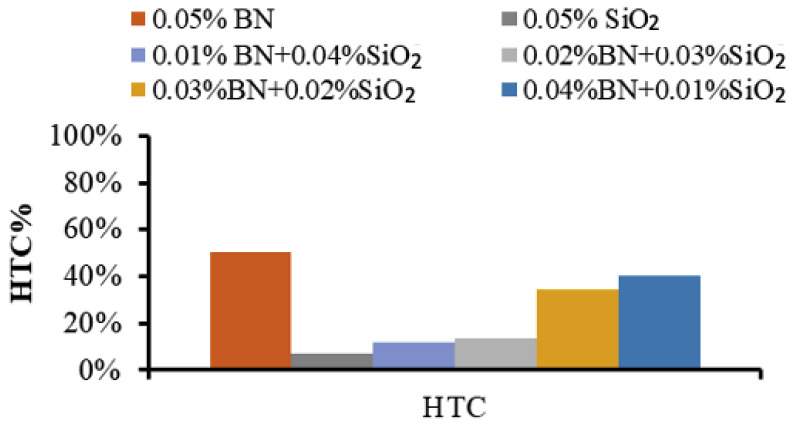
The average enhancement of HTC for the MNFs and HNFs.

On the other hand, the average boiling heat transfer coefficient (HTC) for both types of NF was determined in the nucleate boiling regime of the boiling curves and the results are presented in Figure 6. Like other performance parameters (CHF, BHF), the HTC was found to increase with increasing BN concentration by up to 52% for BN MNF in comparison with that of the BF.

The experimental results (reported in Figure 4, Figure 5 and Figure 6) demonstrate that the preparation of HNF consisting of BN and SiO_2_ NPs resulted in enhanced thermal properties and features for the improvement of the boiling heat transfer performance. In addition, the results confirmed that the MNFs for both BN and SiO_2_ also have good potential for boiling applications. On the other hand, it should be mentioned that the MNF and HNF samples are operated under high-temperature PB conditions, in which their properties have better features in terms of high thermal conductivity and low viscosity. The latter may enhance the movement of NPs and their collision especially for NPs in HNFs, which contain two or more different types of NPs with different properties in terms of density, size, and thermal properties. These factors may cause the enhanced thermal features of HNFs in the PB process. However, the motion phenomena of NPs and their interaction with the BF can be considered key factors for the thermal performance of NFs [29]. The electrophoretic and thermophoretic motions of NPs in NFs can cause chaotic convection to influence the thermal convection of the MNFs and HNFs [30]. Additionally, the modification of the heating surface caused by the deposition of NPs leads to significant advancement in these pool boiling features, i.e., CHF, HTC, and BHF [10,11,31], where the mechanisms of bubble nucleation are enhanced by the increase in the bumps and pits initiated by the deposition of NPs on the heating surfaces [17,32]. Also, the high thermal conductivity of some types of NP may contribute to increased bubble nucleation sites compared to the low thermal conductivity of NPs; hence, the amount of bubble creation is more than the amount of departure, affecting the CHF and BHF values. However, numerous other factors may also affect the behavior of HNFs and MNFs in the PB system, such as the oxidation and the capillary wicking of the surface, which decrease the nucleation site’s space for bubbles [33], and the direction of the heating surface and its geometry [34].

## 6. Conclusions

In this study, the thermal characteristics and performance of mono and hybrid nanofluids of BN and SiO_2_ in pool boiling were experimentally investigated. The experiments were conducted using a validated pool boiling setup ensuring proper conditions and calibration. The boiling features were compared at the same total particle concentration of 0.05 vol.%. The NF samples were prepared in conditions of good stability, and the thermal conductivity was measured. The experimental data were collected for the heat flux as a function of the superheat or excess temperature, and the pool boiling curves were established. Then, the magnitudes of CHF, HTC, and BHF were determined for the HNF and MNF samples. The main conclusions from this study are as follows:

- A good homogenization and stability of the prepared MNF and HNF samples;
- Reasonable enhancements in thermal conductivity for all NFs compared to the BF, increased by the increase in BN NPs’ percentage until reaching the maximum enhancement of about 7% for the BN MNF. The thermal conductivity enhancement of HNFs ranged between 3.2% (at 0.01/0.04 BN/SiO_2_) and 6.5% (at 0.04/0.01 BN/SiO_2_);
- The studied MNF and HNF samples showed significant enhancement in heat transfer performance compared to their BFs, and the performance decreased with the increase in SiO_2_ NPs in the HNF;
- Good enhancements in the CHF and BHF of both MNFs and HNFs were demonstrated. The CHF was enhanced by up to 80% for MNF and up to 69% for HNF at 0.04 vol.% of BN, while the lowest improvement was 48% for the SiO_2_ MNF, compared to the BF.
- The CHF of the HNFs was enhanced by about 69% for BN/SiO_2_ at 0.04/0.01 concentration and 51% for BN/SiO_2_ at 0.01/0.04 concentration;
- The BHF improved as the BN NPs’ concentration increased from 75% for SiO_2_ MNF up to 103% for MNF;
- The HTC improved with increasing BN NP concentration, by up to 52% for BN MNF.

This study provides new findings for prepared HNFs which are useful for the development of advanced thermal management and pool boiling applications.

## Data Availability

Not applicable.

## Nomenclature

BF	Base fluid
BHF	Burnout heat flux (W/m^2^)
BN	Boron Nitride
CHF	Critical heat flux (W/m^2^)
DW	Distilled water
*d*	Wire diameter (mm)
EG	Ethylene Glycol
HNF	Hybrid nanofluid
PB	Pool boiling
*I*	Current (amp)
*L*	Wire length (m)
MNF	Mono nanofluid
NF	Nanofluid
NP	Nanoparticles
q	Heat flux (W/m^2^)
*R*	Electrical resistance (Ohm)
*T_w_*	Wire surface temperature (°C)
*T_sat_*	Saturation temperature of the fluid (°C)
*V*	Voltage (V)
